# Elucidation of the Interactions of Reactive Oxygen Species and Antioxidants in Model Membranes Mimicking Cancer Cells and Normal Cells

**DOI:** 10.3390/membranes12030286

**Published:** 2022-03-01

**Authors:** Geonho Cho, Deborah Lee, Sun Min Kim, Tae-Joon Jeon

**Affiliations:** 1 Department of Biological Sciences and Bioengineering, Inha University, 100, Inha-ro, Michuhol-gu, Incheon 22212, Korea; geonho.cho4607@gmail.com; 2 Department of Biological Engineering, Inha University, 100, Inha-ro, Michuhol-gu, Incheon 22212, Korea; debbielee444@gmail.com; 3 Department of Mechanical Engineering, Inha University, 100, Inha-ro, Michuhol-gu, Incheon 22212, Korea

**Keywords:** biomimetic membranes, lipid membranes, reactive oxygen species, photodynamic therapy, antioxidant

## Abstract

Photosensitizers (PSs) used in photodynamic therapy (PDT) have been developed to selectively destroy tumor cells. However, PSs recurrently reside on the extracellular matrix or affect normal cells in the vicinity, causing side effects. Additionally, the membrane stability of tumor cells and normal cells in the presence of reactive oxygen species (ROS) has not been studied, and the effects of ROS at the membrane level are unclear. In this work, we elucidate the stabilities of model membranes mimicking tumor cells and normal cells in the presence of ROS. The model membranes are constructed according to the degree of saturation in lipids and the bilayers are prepared either in symmetric or asymmetric form. Interestingly, membranes mimicking normal cells are the most vulnerable to ROS, while membranes mimicking tumor cells remain relatively stable. The instability of normal cell membranes may be one cause of the side effects of PDT. Moreover, we also show that ROS levels are controlled by antioxidants, helping to maintain an appropriate amount of ROS when PDT is applied.

## 1. Introduction

Various methods have been developed for cancer therapy, one of which is photodynamic therapy (PDT), where photosensitizers (PSs) accumulate in cancer cells and generate reactive oxygen species (ROS) upon light radiation [1]. ROS damage intracellular components such as DNA, proteins, and lipids, inhibiting cancer cell activity [2,3]. They also oxidize intracellular signaling molecules, leading to various pathways of cell death. In addition, ROS are mainly produced as a byproduct of mitochondria or external UV radiation to the human body [4,5]. Compared to normal cells, cancer cells have high levels of both ROS and antioxidants to maintain a balance between them. For cancer therapy, cell death is induced by breaking the equilibrium by the addition of ROS. These photodynamic treatments causing the apoptotic and necrotic cell death of cancer cells [6,7] have been continuously proposed since skin cancer treatment was first introduced in 1978 [8].

PDT uses biodegradable nanoparticles to deliver PSs [9,10], mainly targeting tumors at a relatively low pH [11,12,13]. However, they may accumulate in nontarget cells, and skin photosensitization occurs as a side effect of residual PS being excited by natural sunlight [14], and cells neighboring the target cells may be subjected to hypoxia and anoxia due to residual PS [15]. A number of studies have focused on the oxidation of internal substances or changes in the signaling of cells caused by ROS interactions [16,17]. In contrast, the structural perturbation attributed to the peroxidation of unsaturated lipids caused by ROS is not fully understood, necessitating further research into the ROS effects in membranes with an abundance of unsaturated lipids.

The plasma membrane is composed of various types of lipids, such as phosphatidylcholine and sphingomyelin [18]. In typical cells, some lipids should not be exposed to the outer layer. For instance, when phosphatidylserine flip-flops to the outer layer, it sends an ‘eat me’ signal to phagocytes and results in apoptosis [19]. Some lipids in the inner leaflet of bilayers are not usually exposed to the extracellular matrix by a transmembrane lipid transporter, flippase, thus forming an asymmetric bilayer in the plasma membrane. The cytoplasmic lipids in the inner leaflet of bilayers are primarily composed of unsaturated lipids, while exoplasmic lipids in the outer leaflet of bilayers are composed of mostly saturated lipids [20]. On the other hand, in cancer cells, cytoplasmic lipids such as phosphatidylserine are exposed to the outer layer due to low flippase activity, forming a symmetric bilayer [21,22]. Therefore, further studies on the ROS interactions with different cell types are needed, since they have different compositions of lipids.

In this study, we created model membranes, including planar bilayers and liposomes, either in symmetric or asymmetric form, to test the ROS interactions of normal cell- and cancer cell-mimicking membranes with different membrane compositions. As a result, the membranes mimicking normal cells were most significantly affected by ROS. Moreover, the reduction in structural damage caused by ROS in the presence of antioxidants was verified, showing that ROS caused structural perturbation to membranes and that ROS levels could be controlled by antioxidants. We expect that our studies will provide more comprehensive information on the effects of ROS on cell membranes in different types of cells with different compositions. In addition, when applied to PDT, the side effects of PDT can be reduced by using an appropriate amount of antioxidants without significantly affecting its therapeutic effect.

## 2. Materials and Methods

### 2.1. Bilayer Formation

The PDMS chamber was manufactured to form a lipid bilayer using aqueous droplets and oil. To limit the contact area of the droplet, a spark generator (DAEDALON, Salem, MA, USA) was used to send 50 sparks to a 0.05 mm-thick PTFE film (Good Fellow, Huntingdon, UK), making an aperture with a diameter of approximately 170 μm, which is the generally established method [23,24]. Then, 0.5% (*w/v*) phospholipid was used in buffer solution (1.0 M KCl, 2 mM KH_2_PO_4_, 8 mM K_2_HPO_4_, and 10 mM EDTA (adjusted to a pH of 7.4)). Sonication (3 mm tip, 21%) was conducted using a probe tip sonicator (VCX500, Sonics & Materials, Inc., Newtown, CT, USA) to achieve complete dissolution. 1,2-dioleoyl-sn-glycero-3-phosphocholine (DOPC; Avanti Polar Lipid, Inc., Alabaster, AL, USA) and 1,2-diphytanoyl-sn-glycero-3-phosphocholine (DPhPC; Avanti Polar Lipid, Inc., Alabaster, AL, USA) were used and mixed at the required ratio. A chamber was created with polydimethylsiloxane (PDMS; Dow Corning, Midland, MA, USA) for ease of manipulation [25], and the PTFE film was inserted. Then, 100 nL of hexadecane (Sigma, St. Louis, MO, USA) was added to the aperture of PTFE, and both chambers were filled with 100 µL of liquid solution to fabricate a lipid layer in the aperture of the PTFE film. This platform was verified by experiments using α-hemolysin (Figures S1 and S2).

### 2.2. Electrical Measurement

An Axopatch 200B patch clamp (Molecular Devices, Sunnyvale, CA, USA) and a DigiData 1440A instrument (Molecular Devices, Sunnyvale, CA, USA) was connected to a 1 mm-thick Ag/AgCl electrode (Alfa Aesar, Ward Hill, MA, USA), and an established method was used [26,27]. The data were acquired via 100 kHz sampling with a low-pass Bessel filter (1 kHz). Since the lipid bilayer exhibited electrical conductivity [28], lipid bilayer formation was confirmed through the increase in capacitance (C).

I = dq/dt(1)

C = εA/d = dq/dV(2)

This equation can be summarized as follows:

I = εA/d × dV/dt(3)

An increase in the absolute value of the current at a constant dV/dt increases the capacitance—that is, the area of the lipid bilayer—leading to the formation of the lipid bilayer. The pipette offset was adjusted to shift the graph toward the negative bias when the conductivity of the lipid bilayer became unstable, and when the droplets fused, the chart dropped to −10,000 pA or less. A triangle wave voltage (±50 mV/20 ms) was used. Data were collected for 30 min, which allowed for a sufficient reaction time, using the Clampfit 10.7 program (Molecular Devices, Sunnyvale, CA, USA); the data were then analyzed using the Clampex 10.7 program (Molecular Devices, Sunnyvale, CA, USA).

### 2.3. ROS Generation

Methylene blue (20 µM; Duksan, Gyeonggi-do, Korea) was used as a photosensitizer. A total of 200 lm of red light, the maximum amount, was irradiated using an ACULED VHL device (PerkinElmer, Waltham, MA, USA) to produce singlet oxygen [29,30].

### 2.4. Liposome Generation

The inverted emulsion method was used to generate asymmetric liposomes [31] (Figure S3). Two types of aqueous solutions were prepared. Briefly, 0.2 mM calcein, 25 mM HEPES-NaOH (pH 7), and 475 mM sucrose were added to the inner aqueous solution, while 25 mM HEPES-NaOH (pH 7) and 475 mM glucose were added to the reservoir solution. Next, 100 µL of the internal aquatic solution was added to 800 µL of mineral oil (lipid 0.1% *w/v*), sonicated in an icebox for 4 min using a probe tip sonicator (2 s/2 s pulse), and then incubated for 5 min. A 1.5 mL microcentrifuge tube was coated with bovine serum albumin (BSA; Sigma, St. Louis, MO, USA). Then, 500 µL of reservoir solution was added, followed by 400 μL of emulsion solution, and the mixture was incubated for 10 min. After centrifugation at 16,000 g for 15 min at 8 °C, the supernatant was removed. Then, 1.1 mL of reservoir solution was added again and pipetted gently to disperse the pellet nicely. Only 1 mL of resolution reservoir at the bottom was collected to remove the remaining oil emulsion. All liposomes were refrigerated and used within 24 h. In the case of asymmetric liposomes, dodecane (Sigma, St. Louis, MO, USA) (0.1% *w/v* lipid) was additionally used when producing the emulsion solution. Finally, 500 µL of the reservoir solution, 400 µL of mineral oil (0.1% *w/v* lipid), and 400 µL of the emulsion were added sequentially and centrifuged until homogeneous.

### 2.5. Liposome Quenching Experiment

Liposome solution, 2 mM calcein quencher [32] FeCl_3_ (Sigma, St. Louis, MO, USA), and 20 µM methylene blue were added to reach a total of 1 mL in a 1.5 mL cuvette. The initial fluorescence intensity was measured at Ex/Em = 495/515 nm using a spectrophotometer (RF-5301PC, Shimadzu Europe, Institute, Arlington, TX, USA). Red light was irradiated at 10 °C for 30 min, and the fluorescence intensity was measured.

### 2.6. Antioxidant Test

L-glutathione (Sigma, St. Louis, MO, USA) and ascorbate (Sigma, St. Louis, MO, USA) were used as water-soluble antioxidants, and α-tocopherol (Sigma, St. Louis, MO, USA) and β-carotene (Sigma, St. Louis, MO, USA) were used as fat-soluble antioxidants. Briefly, 200 μM water-soluble antioxidants were placed on either the inside or outside of the liposomes. Fat-soluble antioxidants were used in mineral oil and emulsion solution in a mole ratio of lipid:cholesterol:antioxidant = 0.7:0.295:0.005. Additionally, 20 µM of methylene blue was measured by the same method as the liposome permeability analysis.

## 3. Results and Discussion

PDT is a cancer therapy in which the generated ROS oxidize various substances inside the cell. ROS also oxidize and change the structure of lipids in cell membranes, changing the permeability of the membrane [33]. Due to the differences in the membrane compositions of normal and cancer cells, the ROS effects of the membranes and their permeability would vary in response to ROS. To observe the different membrane interactions of ROS, we carried out experiments on the membrane stability and permeability in the presence of ROS generated by photosensitizers.

To mimic the lipid composition of cell membranes for normal cells and cancer cells, we constructed model lipid bilayers as follows. Most lipids in the cytoplasmic leaflet of membranes are unsaturated, and those in the exoplasmic leaflet are saturated. Hence, the model membranes contain unsaturated lipids, DOPCs, in the inner leaflet of lipid bilayers, and saturated lipids, DPhPCs, in the outer leaflet of lipid bilayers. Due to the low flippase activities in cancer cells, the lipid compositions remain similar in both layers, resulting in a symmetric bilayer; thus, the model bilayers are formed of 1:1 DOPC and DPhPC mixtures. Although oxidation may not influence the head groups more than the tails of the lipids [34], the same head group, phosphocholine (PC), was used for all experiments to minimize the head group effects. In this study, model membranes with the compositions described above were made in two different forms—planar lipid bilayers and liposomes—to elucidate the interactions of singlet oxygen with each lipid composition.

### 3.1. Planar Lipid Bilayer

A planar lipid bilayer is mainly used to measure the movements of ions/small molecules through the aperture of channel proteins/nanopores in the lipid bilayer. Electric currents are not measurable before a bilayer is formed due to the nonconducting solvent between the two leaflet of lipid layers. When the bilayer is formed, electric current flows slightly through the bilayer. When a small aperture forms in the bilayer, the current will increase. Accordingly, the current will be saturated if the bilayer ruptures and the electrodes in both chambers have no resistance from the bilayer. We applied a triangle wave through the bilayer to verify the membrane formation and stability. When the leakage current increases, the signal will gradually increase to a positive or negative bias. To monitor the leakage of the bilayers, we adjusted the offset toward a negative bias so that we could observe leakage when the signal shifted toward a negative bias. When the membrane ruptures, the signal will be saturated, indicating that the membrane no longer remains intact. The stability of bilayers may vary according to their unsaturated lipid content because ROS may react with unsaturated lipids. Various lipid compositions were tested in this work to elucidate the interactions of the model cell membranes in the presence of ROS.

#### 3.1.1. Symmetric Bilayer

**Figure 1:**
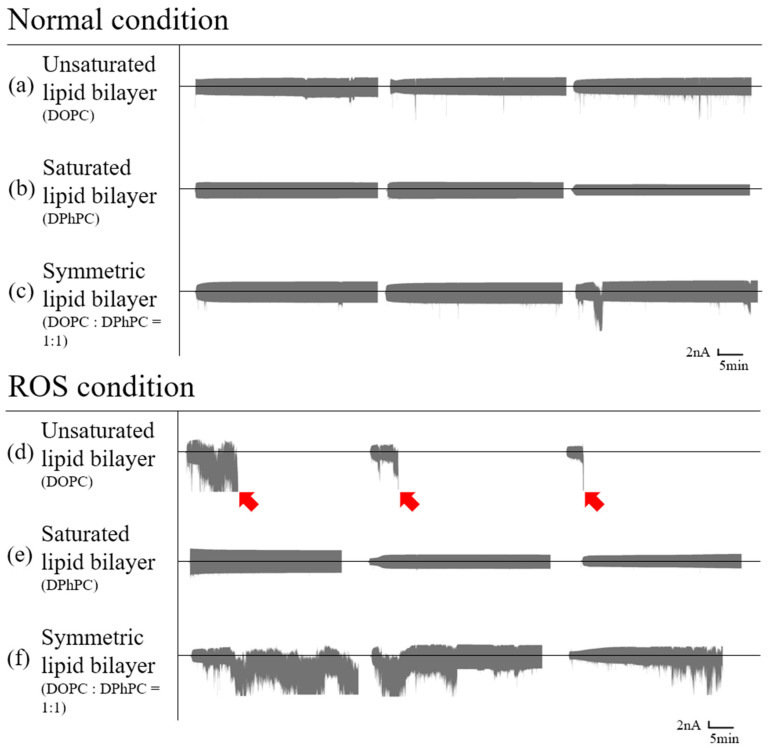
Symmetric planar lipid bilayer electric current graph with a triangle wave voltage; the middle line of each chart is zero amperes (Figure S4). Red arrows indicate that the droplets no longer remain intact. Lipid bilayer in the absence of ROS (unsaturated; (**a**), saturated; (**b**), mixed; (**c**)) and the presence of ROS (unsaturated; (**d**), saturated; (**e**), mixed; (**f**)).

Symmetric lipid bilayers were formed with only DOPC and DPhPC for control experiments and a 1:1 mixture of DOPC and DPhPC. Each model membrane was then measured and analyzed with/without ROS. To generate singlet oxygen, methylene blue was added into both chambers, and red light was used for illumination upon the formation of a bilayer. In the absence of ROS, three types of bilayers with differences in the compositions of lipids remained intact (Figure 1a–c). In the presence of ROS, bilayers with only saturated lipids remained intact for more than 30 min (Figure 1e), whereas the stability of bilayers with unsaturated lipids decreased. This is attributed to the peroxidation of carbon-carbon double bonds in the tail of unsaturated lipids, causing the lipid molecules to stretch and shrink horizontally; thus, the hydrophobic interactions of the unsaturated lipid tail were decreased due to peroxidation [35,36]. Interestingly, symmetric bilayers with a 1:1 mixture of saturated and unsaturated lipids appeared unstable and ruptured (Figure 1f), while bilayers with only unsaturated lipids ruptured all times upon the addition of ROS (Figure 1d).

#### 3.1.2. Asymmetric Bilayer

**Figure 2:**
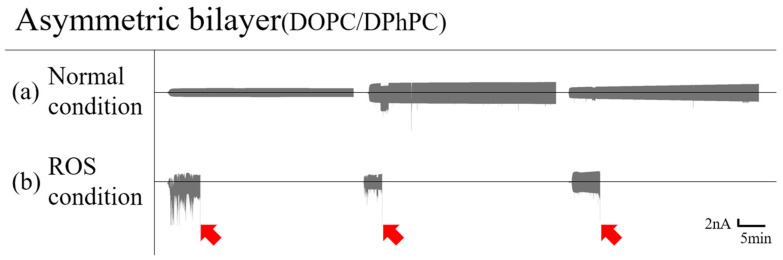
Asymmetric planar lipid bilayer electric current graph with a triangle wave voltage; the middle line of each graph is zero amperes. Red arrows indicate that the droplets no longer remain intact. Asymmetric bilayer in the absence of ROS (**a**) and in the presence of ROS (**b**).

Most lipids in the cytoplasmic leaflet of normal cell membranes are unsaturated, and those in the exoplasmic leaflet of lipids are saturated. The lipid bilayers mimicking normal cell membranes are made in an asymmetric form containing DOPC in one leaflet and DPhPC in the other leaflet. Singlet oxygen made inside the cell can diffuse across the membrane by passive diffusion [37]. Similarly, in our system, the stability of the bilayer can be affected when singlet oxygen permeates through the DPhPC layer and reaches the DOPC layer. In the absence of ROS, the asymmetric bilayer remained intact (Figure 2a) while the bilayers ruptured, and the current signals were accordingly saturated in the presence of ROS. This observation is also consistent in the symmetric bilayer, where the bilayer with unsaturated lipids becomes unstable due to the peroxidation of the unsaturated lipids in the presence of ROS (Figure 2b).

### 3.2. ROS Effect on Liposomes

In addition to the membrane stability tests with planar bilayers, we employed liposomes in the different compositions of lipids to further study membrane permeability and stability in a more quantitative manner, where ions permeated through the membrane were measured with and without ROS present in solution. To test ion permeability, calcein as a fluorescence molecule was encapsulated inside of the liposomes, and Fe^3+^ ions as a fluorescence quencher were added outside of the liposomes. When ROS peroxidize unsaturated lipids, the lipids in the membrane become loosely held or rupture due to structural changes, increasing the permeability of ions through the membrane. The membrane permeability will vary according to the stability of liposomes in different compositions of lipids, and ROS effects on the membranes can be quantitatively measured.

#### 3.2.1. ROS Effect on Several Compositions of Liposomes

**Figure 3:**
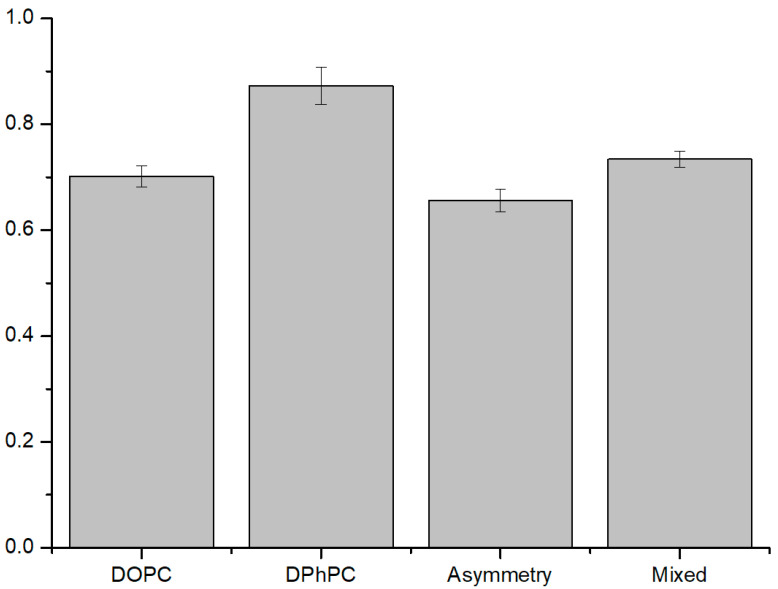
Graph of liposome stability in several compositions in the presence of ROS. It is a value of change in fluorescence intensity (after light illumination/before light illumination) (N = 8). The smaller the value is, the more unstable it is. Symmetric liposomes of DPhPC were the most stable, and asymmetric liposomes were the most unstable. Mixed liposomes with DPhPC and DOPC in symmetric form were more stable than with DOPC only liposomes, and DOPC only liposomes were more stable than asymmetric liposomes (Table S1).

We created symmetric liposomes mimicking cancer cell membranes formed with a 1:1 mixture of DOPC and DPhPC and asymmetric liposomes mimicking normal cell membranes having an inner leaflet with DOPC and outer with DPhPC resembling the normal cell membrane. In addition, liposomes composed only of DOPC and DPhPC were used as controls. Although ROS does not affect the lipid tail of DPhPC, liposomes made only with DPhPC showed fluorescence values less than the theoretical value of 1 due to the instability of the liposome itself (Figure 3). However, it had the smallest ROS effect compared to other compositions of liposomes. The liposomes with asymmetric membranes were significantly affected by ROS, showing the lowest fluorescence values. Comparing the stabilities of liposomes with DOPC only and asymmetric membranes, the former liposomes with symmetric membranes only with unsaturated lipids showed a higher fluorescence value than the latter liposomes with asymmetric membranes, indicating that lipid content with unsaturated lipids in the inner leaflet has a greater effect on the stability of the liposome. Interestingly, the liposomes with a 1:1 mixture of DOPC and DPhPC were the most stable among the liposomes with unsaturated lipids, resulting in relatively minor leakage being caused by ROS because the peroxidized lipid tail fractions may have cholesterol-like effects [34], showing that the membranes with a mixture of unsaturated lipids and saturated lipids are the most stable against structural deformation in the presence of ROS.

#### 3.2.2. Antioxidant Effect on ROS Condition

Antioxidants were used to determine whether ROS were the main cause of membrane leakage in the ROS experiment. Moreover, antioxidants can be used to properly regulate the amount of ROS generated in PDTs to minimize the side effects resulting from singlet oxygen.

**Figure 4:**
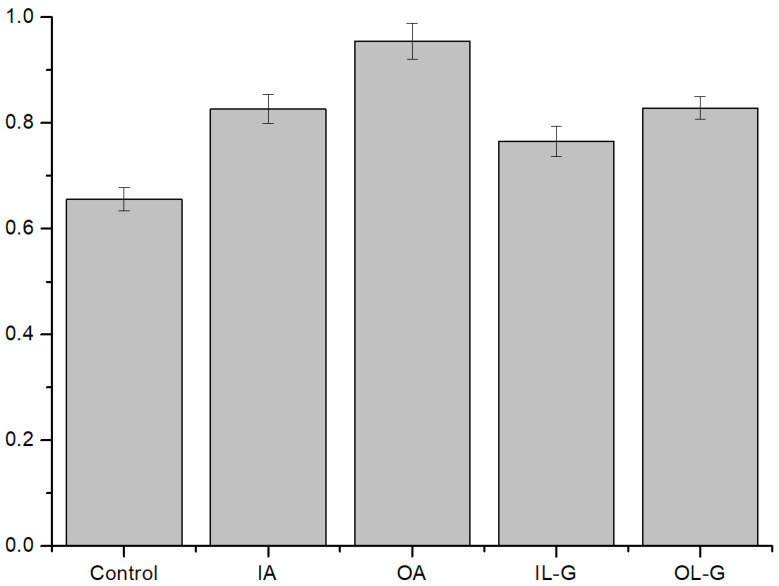
Comparison of the activities of two types of water-soluble antioxidants. There are four kinds of liposomes: inner ascorbate (IA), outer ascorbate (OA), inner L-glutathione (IL-G), and outer L-glutathione (OL-G). Inner refers to inside the liposome, while outer is outside the liposome. The concentration of both the inside and outside antioxidants was 200 µM. The ratio of the fluorescence intensity difference was analyzed (after light illumination/before light illumination) (N = 8). The smaller the value is, the more unstable it is. The control was an asymmetric liposome. Outer ascorbate showed the highest ROS scavenging effect, followed by inner ascorbate, outer L-glutathione, and inner L-glutathione. (Table S1).

Ascorbate is an antioxidant known as vitamin C, and L-glutathione is an intracellular antioxidant found in plants, animals, fungi, etc. ROS in the presence of antioxidants are scavenged; thus, the peroxidation of lipids decreases. We used both ascorbate and L-glutathione to observe the activities of antioxidants when added inside and outside of the liposomes. When antioxidants were placed outside the liposomes, they showed the considerable stability of the membranes by ROS uptake, while the efficiency of ROS uptake was not as effective when the antioxidants were added inside of the liposomes (Figure 4). Given the comparison between ascorbate and L-glutathione, ascorbate had a great effect when added both inside and outside of liposomes, showing that the level of singlet oxygen could be more efficiently controlled by ascorbate in our experiments, similar to previously reported results [38].

**Figure 5:**
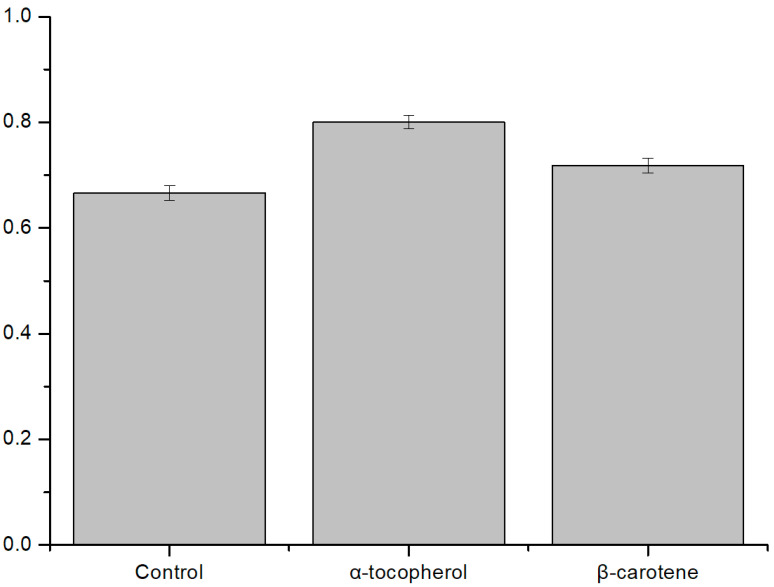
Comparison of the activities of two types of fat-soluble antioxidants. α-tocopherol and β-carotene were used at a molar ratio of 0.05% of the total concentration. The ratio of the fluorescence intensity difference was analyzed (after light illumination/before light illumination) (N = 8). The smaller the value is, the more unstable it is. The control was an asymmetric liposome. α-tocopherol has a greater ROS scavenging effect than β-carotene (Table S1).

We also tested the fat-soluble antioxidants α-tocopherol and β-carotene. α-tocopherol is an antioxidant well known as vitamin E, and β-carotene is one of the pigments of plants and is a precursor of vitamin A with antioxidant properties. These fat-soluble antioxidants are usually present within the membranes of liposomes. Both types of fat-soluble antioxidants showed increased membrane stability of liposomes in the presence of antioxidants by ROS uptake, as in previous experiments with water-soluble antioxidants (Figure 5). In these experiments with two different fat-soluble antioxidants, α-tocopherol showed a greater efficiency of ROS uptake than β-carotene.

In this study, we learned that symmetric membranes with a mixture of unsaturated lipids and saturated lipids are more resistant to ROS in terms of membrane stability than asymmetric membranes with unsaturated lipids in the inner leaflet of bilayers that are more vulnerable to singlet oxygen. Our results indicate that the side effects of PDT can be attributed to the membrane stability of normal cells and may be problematic, since the membrane composition of normal cells is more susceptible to ROS. Additionally, the level of ROS can be efficiently controlled by antioxidants to prevent intracellular oxidation and lower membrane damage caused by ROS.

## 4. Conclusions

We made model membranes mimicking normal cells and cancer cells to elucidate membrane stability in the presence of ROS and to determine how ROS levels could be controlled by antioxidants. The normal cell-mimicking membranes in the form of planar bilayers and liposomes appeared most vulnerable to ROS, making membranes unstable in a short time, which could be explained by photosensitivity [39] or other side effects [40,41] commonly observed during PDT; notably, PDT could be more effective on normal cell membranes. On the other hand, cancer cell-mimicking membranes remained stable for a longer time in the presence of ROS. Although several mechanisms have been noted to underlie this structural deformation in different studies, such as cytoskeleton and membrane interactions [42], deformation through cyclic hypoxia [43], and intercellular damage [44], our results support the notion that the structural perturbation of membranes is induced by lipid peroxidation in the presence of ROS [45,46]. Therefore, ROS effects on the lipid bilayers of normal cell membranes should be considered when applying photodynamic therapy, radiotherapy, chemotherapy, etc., where ROS are used or generated. Therapies associated with a small amount of antioxidants can be one option to maintain an appropriate level of ROS, thus minimizing the side effects of ROS. We believe that our results will contribute to the design of safer cancer therapies where ROS are applied and further the understanding of the molecular interactions of ROS in cell membranes.

## Data Availability

Not applicable.

## Supplementary Materials

The following supporting information can be downloaded at https://www.mdpi.com/article/10.3390/membranes12030286/s1. Figure S1: PDMS-PTFE Chamber. PTFE film inserted in PDMS chamber. W × D × H; 20 × 20 × 16 mm^3^. Figure S2: PDMS-PTFE chamber tested with α-Hemolysin (0.7 μM). The formation of the bilayer was confirmed using triangle wave. By the increase of electric current, it was confirmed that α-Hemolysin was attached to the bilayer. Figure S3: Liposomes generated using the inverted emulsion method. Asymmetric form was confirmed using NBD (Ex/Em = 470/536 nm). Left: NBD in inner leaflet, Right: NBD in outer leaflet. Green, Red: liposomes with NBD. Yellow, Blue: liposomes with NBD + NBD quencher. Pink: liposomes with NBD + NBD quencher + SDS. Figure S4: How to understand the graph, and triangle wave that we used. Pipette offset was 8.56. Table S1: Liposome experiments raw data.
