# Supplementary material for: Elucidation of the Interactions of Reactive Oxygen Species and Antioxidants in Model Membranes Mimicking Cancer Cells and Normal Cells
Source: Membranes (Basel). 2022 Mar 1;12(3):286. doi: 10.3390/membranes12030286 (PMC8949560; doi:10.3390/membranes12030286)
Supplement: Supplementary file 1 [file membranes-12-00286-s001.zip › membranes-1599394-supplementary.pdf]

membranes

MDPI

Article

Elucidation of the Interactions of Reactive Oxygen Species and Antioxidants in Model Membranes Mimicking Cancer Cells and Normal Cells

Geonho Cho [1], Deborah Lee [2], Sun Min Kim [1,3,*] and Tae-Joon Jeon [1,2,*]

[1] Department of Biological Sciences and Bioengineering, Inha University, 100, Inha-ro, Michuhol-gu, Incheon 22212, Korea; geonho.cho4607@gmail.com
[2] Department of Biological Engineering, Inha University, 100, Inha-ro, Michuhol-gu, Incheon 22212, Korea; debbielee444@gmail.com
[3] Department of Mechanical Engineering, Inha University, 100, Inha-ro, Michuhol-gu, Incheon 22212, Korea
* Correspondence: sunmk@inha.ac.kr (S.M.K.); tjjeon@inha.ac.kr (T.-J.J.)

Citation: Cho, G.; Lee, D.; Kim, S.M.; Jeon, T.-J. Elucidation of the Interactions of Reactive Oxygen Species and Antioxidants in Model Membranes Mimicking Cancer Cells and Normal Cells. *Membranes* **2022**, *12*, 286. https://doi.org/10.3390/membranes12030286

Academic Editor: Masanao Kinoshita

Received: 31 January 2022
Accepted: 24 February 2022
Published: date

Publisher's Note: MDPI stays neutral with regard to jurisdictional claims in published maps and institutional affiliations.

Copyright: © 2022 by the authors. Submitted for possible open access publication under the terms and conditions of the Creative Commons Attribution (CC BY) license (https://creativecommons.org/licenses/by/4.0/).

Figure S1: PDMS-PTFE Chamber. PTFE film inserted in PDMS chamber. W*D*H; 20*20*16 mm^3

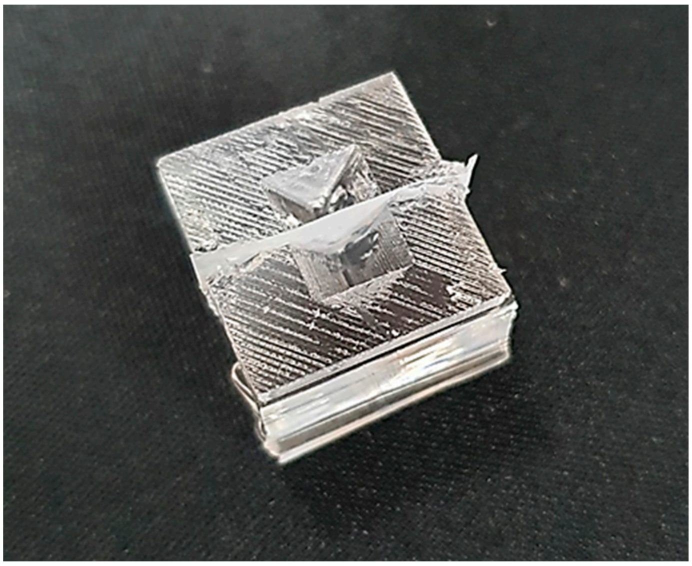

Figure S2: PDMS-PTFE chamber tested with α-Hemolysin(0.7 μM). The formation of the bilayer was confirmed using triangle wave. By the increase of electric current, it was confirmed that α-Hemolysin was attached to the bilayer.

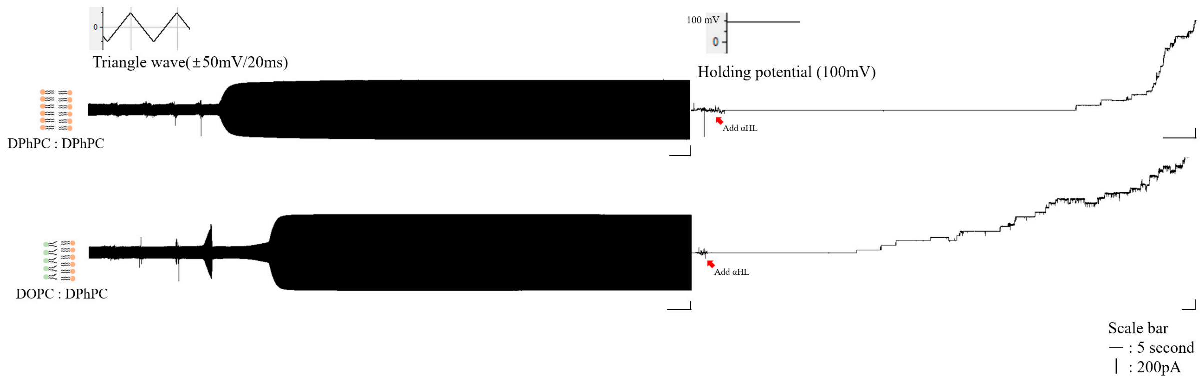

Figure S3: Liposomes generated using the inverted emulsion method. Asymmetric form was confirmed using NBD(Ex/Em=470/536nm). Left: NBD in inner leaflet, Right: NBD in outer leaflet

Green, Red: liposomes with NBD

Yellow, Blue: liposomes with NBD + NBD quencher

Pink: liposomes with NBD + NBD quencher + SDS

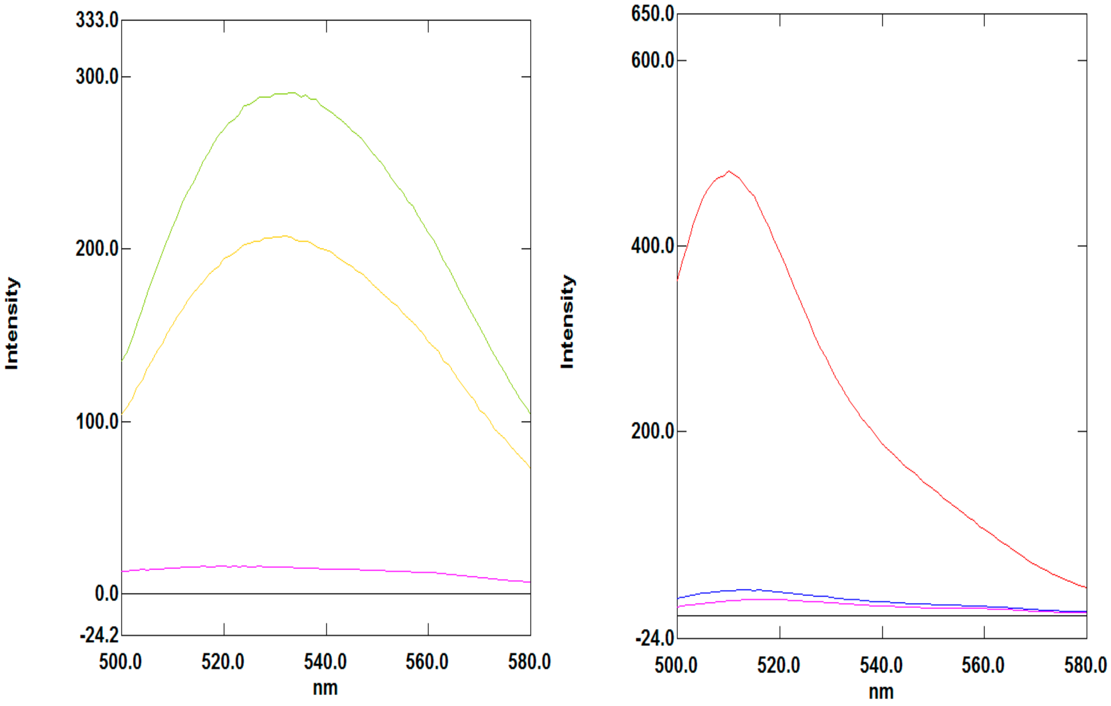

Figure S4: How to understand the graph, and triangle wave that we used. Pipette offset was 8.56.

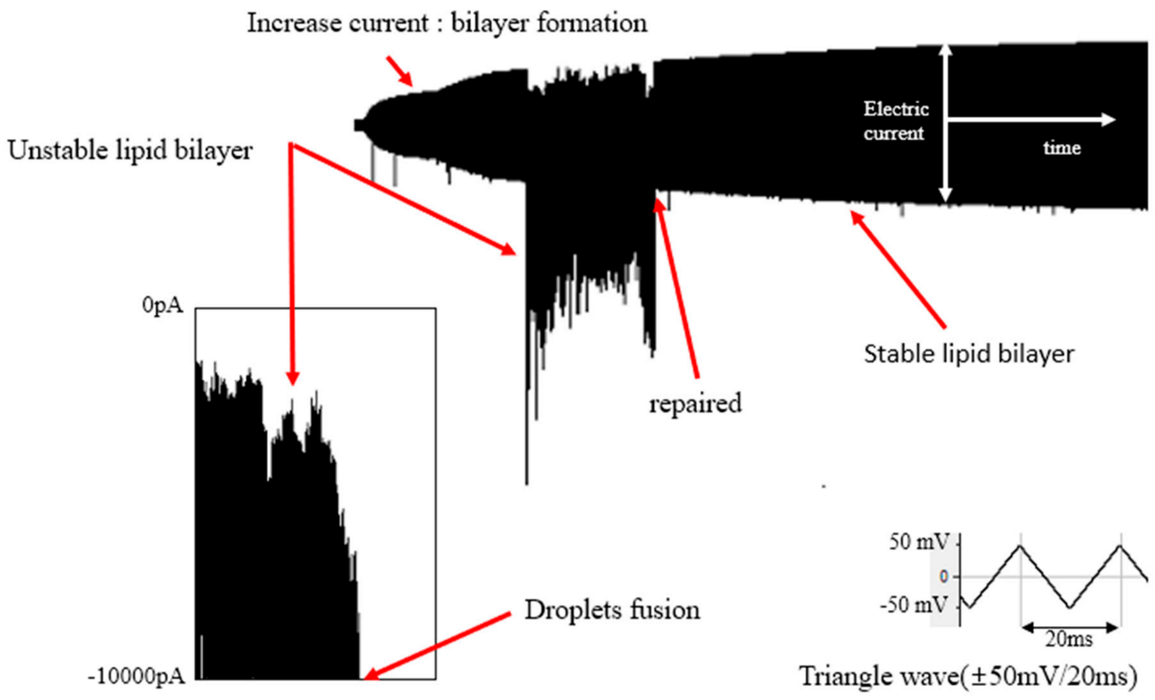

Table S1: Liposome experiments raw data

DOPC	Before	After	A/B	DPhPC	Before	After	A/B	Asymmetry	Before	After	A/B	Mixed	Before	After	A/B
	55.4	37.2	0.671		52.6	42.2	0.802		67.7	43.9	0.648		55.4	37.8	0.682
	59.2	38.7	0.654		47.2	43	0.911		61.2	36.8	0.601		49.9	35.8	0.717
	52.6	35.4	0.673		53	37.2	0.702		54.2	41.7	0.769		53.4	36.3	0.680
	51.6	33.2	0.643		53.8	47.5	0.883		70.9	39.7	0.560		54.6	41.1	0.753
	54.6	43.3	0.793		55.8	57.9	1.038		58.9	40.3	0.684		49.9	35.3	0.707
	53.8	42.1	0.783		64.4	53.8	0.835		55.3	37.9	0.685		54.1	41.8	0.773
	60.6	41.3	0.682		59.1	55.2	0.934		59.1	37.7	0.638		48.2	38.2	0.793
	56.7	40.6	0.716		62.4	54.6	0.875		59	39	0.661		48.8	37.5	0.768
Avg.			0.702				0.873				0.656				0.734

inner-ascorbate	Before	After	A/B	outer-ascorbate	Before	After	A/B	Inner L-Glutathione	Before	After	A/B	Outer L-Glutathione	Before	After	A/B
	968.9	849.8	0.877		18.5	18.5	1.000		27.8	19.8	0.712		20.9	17.5	0.837
	853.9	786.5	0.921		20.3	17.6	0.867		24.8	17.5	0.706		21.4	17.6	0.822
	950.7	775	0.815		18.2	18.8	1.033		26.8	18.5	0.690		21.9	16.4	0.749
	866.7	805	0.929		19.1	16.4	0.859		25.6	17	0.664		20.9	18.8	0.900
	664.7	544.1	0.819		19.1	18.9	0.990		14.5	12	0.828		21.8	15.9	0.729
	837.5	596.7	0.712		18.7	16.6	0.888		14.4	11.6	0.806		20.8	17.2	0.827
	719.3	557.7	0.775		16.6	18.7	1.127		12.1	10.4	0.860		18.6	16.3	0.876
	853.9	651.7	0.763		20.8	18.2	0.875		14.3	12.3	0.860		19.1	16.9	0.885
Avg.			0.826				0.955				0.766				0.828

Control	Before	After	A/B	α-tocopherol	Before	After	A/B	β-carotene	Before	After	A/B
	107.1	69.4	0.648		55.1	41.2	0.748		57.8	42.6	0.737
	108.1	75.9	0.702		54.2	44.3	0.817		62	43	0.694
	112.3	70.8	0.630		52.2	42.4	0.812		55.4	43.6	0.787

	119.5	72	0.603		53.9	41.1	0.763		60.1	43.3	0.720
	80.1	52.9	0.660		41.2	33.3	0.808		54.1	35.9	0.664
	80.3	57.5	0.716		41.2	31.9	0.774		48.2	36.1	0.749
	75	53.1	0.708		42	34.8	0.829		52.5	36.5	0.695
	78.7	51.9	0.659		43.3	36.9	0.852		53.5	37.4	0.699
Avg.			0.666				0.800				0.718
